# Unguided Computer-Assisted Self-Help Interventions Without Human Contact in Patients With Obsessive-Compulsive Disorder: Systematic Review and Meta-analysis

**DOI:** 10.2196/35940

**Published:** 2022-04-21

**Authors:** Hissei Imai, Aran Tajika, Hisashi Narita, Naoki Yoshinaga, Kenichi Kimura, Hideki Nakamura, Nozomi Takeshima, Yu Hayasaka, Yusuke Ogawa, Toshi Furukawa

**Affiliations:** 1 Department of Health Promotion and Human Behavior Graduate School of Medicine, School of Public Health Kyoto University Kyoto Japan; 2 Department of Psychiatry Graduate School of Medicine Hokkaido University Sapporo Japan; 3 Department of Neurology Graduate School of Medicine Hokkaido University Sapporo Japan; 4 School of Nursing Faculty of Medicine University of Miyazaki Miyazaki Japan; 5 Department of Psychiatry Hakodate Watanabe Hospital Hakodate Japan; 6 Department of Cognitive Behavioral Physiology Graduate School of Medicine Chiba University Chiba Japan; 7 Department of Healthcare Epidemiology Graduate School of Medicine, School of Public Health Kyoto University Kyoto Japan

**Keywords:** randomized controlled trial, RCT, information technology, psychotherapy, treatment adherence, anxiety disorder, anxiety, OCD, obsessive-compulsive disorder, systematic review, meta-analysis, mental health, computer-assisted, therapy, efficacy, acceptability, eHealth, mental illness

## Abstract

**Background:**

Computer-assisted treatment may reduce therapist contact and costs and promote client participation. This meta-analysis examined the efficacy and acceptability of an unguided computer-assisted therapy in patients with obsessive-compulsive disorder (OCD) compared with a waiting list or attention placebo.

**Objective:**

This study aimed to evaluate the effectiveness and adherence of computer-assisted self-help treatment without human contact in patients with OCD using a systematic review and meta-analysis approach.

**Methods:**

Randomized controlled trials with participants primarily diagnosed with OCD by health professionals with clinically significant OCD symptoms as measured with validated scales were included. The interventions included self-help treatment through the internet, computers, and smartphones. We excluded interventions that used human contact. We conducted a search on PubMed, Cochrane Central Register of Controlled Trials, EMBASE, World Health Organization International Clinical Trials Registry Platform, and ClinicalTrials.gov, as well as the reference lists of the included studies. The risk of bias was evaluated using version 2 of the Cochrane risk-of-bias tool for randomized trials. We calculated the standardized mean differences for continuous outcomes and risk ratios for dichotomous outcomes. The primary outcomes were short-term improvement of OCD symptoms measured by validated scales and dropout for any reason.

**Results:**

We included 11 randomized controlled trials with a total of 983 participants. The results indicated that unguided computer-assisted self-help therapy was significantly more effective than a waiting list or psychological placebo (standard mean difference −0.47, 95% CI −0.73 to −0.22). Unguided computer-assisted self-help therapy had more dropouts for any reason than waiting list or psychological placebo (risk ratio 1.98, 95% CI 1.21 to 3.23). However, the quality of evidence was very low because of the risk of bias and inconsistent results among the included studies. The subgroup analysis showed that exposure response and prevention and an intervention duration of more than 4 weeks strengthen the efficacy without worsening acceptability. Only a few studies have examined the interaction between participants and systems, and no study has used gamification. Most researchers only used text-based interventions, and no study has used a mobile device. The overall risk of bias of the included studies was high and the heterogeneity of results was moderate to considerable.

**Conclusions:**

Unguided computer-assisted self-help therapy for OCD is effective compared with waiting lists or psychological placebo. An exposure response and prevention component and intervention duration of more than 4 weeks may strengthen the efficacy without worsening the acceptability of the therapy.

**Trial Registration:**

PROSPERO (International Prospective Register of Systematic Reviews) CRD42021264644; https://www.crd.york.ac.uk/prospero/display_record.php?RecordID=264644

## Introduction

Obsessive-compulsive disorder (OCD) is characterized by intrusive and unwanted thoughts, urges, or images and repetitive behavior or mental acts [1]. Affected patients try to ignore or suppress OCD symptoms; however, it impairs their ability to carry out daily life activities and deteriorates their quality of life (QOL). The median prevalence of OCD in 1 year was 1.0% (IQR 0.6% to 2.0%), and the cost associated with OCD was estimated as $10.6 billion per year in the United States alone [2].

The treatment of OCD involves psychotherapy and pharmacotherapy; however, psychotherapy may be a better treatment for OCD than pharmacotherapy [3]. Patients with psychiatric disorders prefer psychotherapy over pharmacotherapy [4]. Therefore, guidelines such as the National Institute for Health and Care Excellence recommend cognitive behavioral therapy (CBT) as the initial treatment for OCD [5].

Despite the presence of guidelines for the treatment of OCD, there are hindrances to therapy such as poor help-seeking behavior and inaccessible treatment. A study showed that more than half of patients with OCD have not received treatment [6]. Barriers to seeking treatment include shame about the symptoms or about asking for treatment, lack of knowledge regarding resources, and treatment-related inconveniences [7].

Computer and internet-based treatment is a promising way to overcome these barriers. It can reduce therapist contact and costs and promote client participation in therapies conducted in a nonclinical setting [8]. Successful internet-based interventions include engagement by the user for weeks to months. Examples are interactive elements such as prompted personalized feedback, self-monitoring, and assignment [9]. All the interventions contain educational materials and frequently use cognitive behavioral elements [9]. More specifically, computerized therapy for OCD often includes psychoeducation, cognitive elements, and exposure and response prevention (ERP) [10].

Systematic reviews were conducted on studies including computer-assisted treatment for OCD, but there were limitations; Pearcy et al [11] examined self-help intervention against OCD, but they included quasi-randomized controlled trials (RCTs); Firth et al [12] examined smartphone interventions, but the focus was on anxiety disorder as a whole; and Tumur et al [10] examined computer-assisted CBT for OCD, but it included only one substantial program. These studies need to be updated since the research was conducted in 2015, 2016, and 2004, respectively, and particularly because the rate of publication on digital health has been increasing rapidly since 2015 [13].

Excluding therapist contact and therapy using information technology will improve access to treatment. However, the effectiveness and adherence of computer-assisted interventions without human contact has not been examined through systematic review and meta-analysis. Additionally, the influence of several variables should be examined. For example, ERP is an effective and widely used component for OCD treatment, but therapist assistance is suggested to increase its effectiveness [14,15], the duration or number of sessions attended may be influential factors in psychotherapy [16,17], and the effect of device characteristics and their contents, such as gamification and interaction, have not yet been established [18,19].

Therefore, this study aimed to evaluate the effectiveness and adherence of computer-assisted self-help treatment without human contact in patients with OCD using a systematic review and meta-analysis approach.

## Methods

### Selection Criteria

RCTs with participants primarily diagnosed with OCD according to the *Diagnostic and Statistical Manual of Mental Disorders, Fourth Edition, Text Revision* (DSM-IV-TR)*, and Fifth Edition* (DSM-5) and *International Classification of Diseases, Tenth Revision*, and those who were diagnosed by health professionals and had clinically significant OCD symptoms as measured with validated scales were included. Patients of any age and comorbidities were included. The interventions included self-help treatment through the internet, computers, and smartphones. Sending a digital treatment manual by email was also included because it uses the computer and internet. We excluded interventions that used human contact (except for technical support). We defined human contact as interventions with face-to-face support or interaction with humans on the internet or telephone; self-help means that participants conduct treatment without human contact. Comparisons were made with respect to a placebo condition, including a psychological placebo and a waiting list. Any cotreatment was allowed if it was provided equally to both groups.

### Ethics Approval

We followed the PRISMA (Preferred Reporting Items for Systematic Reviews and Meta-Analyses) guidelines [20; Multimedia Appendix 1]. The protocol for this systematic review was registered at PROSPERO (International Prospective Register of Systematic Reviews) [CRD42021264644].

### Outcomes

The primary outcomes were short-term subjective improvement of OCD symptoms as measured by validated scales such as the Yale-Brown Obsessive-Compulsive Scale (Y-BOCS) and the Obsessive-Compulsive Inventory–Revised (OCI-R) and dropout for any reason at posttreatment. We defined short term as a period of 6 months.

Secondary outcomes were short-term response rate defined by validated scales and anxiety, depression, and QOL measured by validated scales. These outcomes measured at long term were also included in the secondary outcomes. We defined long term as a period greater than 6 months and gave priority to the longest end point.

### Search Methods

We conducted a search on July 28, 2021, in PubMed, Cochrane Central Register of Controlled Trials, EMBASE, World Health Organization International Clinical Trials Registry Platform, and ClinicalTrials.gov, as well as the reference lists of the included studies (Multimedia Appendix 2). We conducted a grey literature search in devices@FDA, a catalog of cleared and approved medical device information. We applied no search restrictions on date, language, or publication status.

### Selection of Studies and Data Extraction

Two authors independently examined the titles and abstracts of the references identified in the search and included them in the second screening if at least 1 author judged them to be included. We then obtained and examined the full text of the included studies using the first screening process. Finally, we included the studies that both reviewers felt should be included. If the 2 authors disagreed after a discussion, a third author was consulted to make a decision. We conducted data extraction in the same way as in the second screening process. We contacted the authors of the studies to obtain additional data or further clarification if needed.

### Measurement of Outcomes

We calculated the standardized mean differences (SMDs) and their 95% confidence intervals for continuous outcomes and risk ratios and their 95% confidence intervals for dichotomous outcomes. We used a random effects model.

### Assessment of Risk of Bias

**Figure 1:**
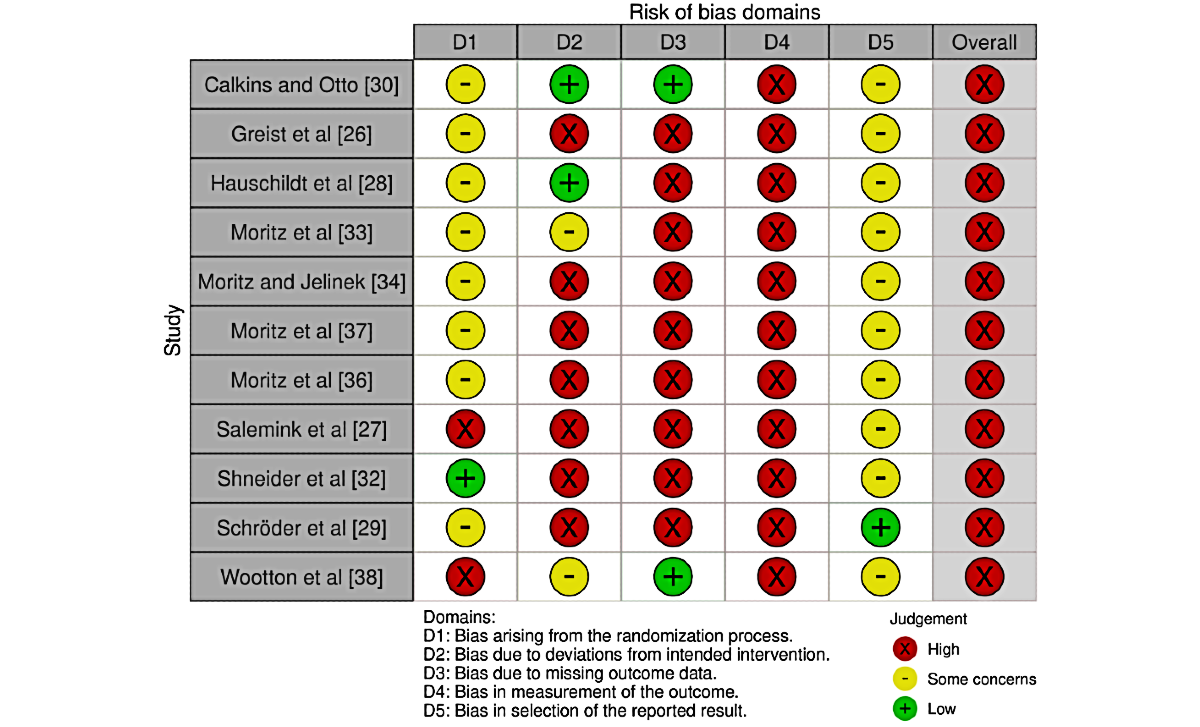
Risk of bias in included studies.

The risk of bias was evaluated using version 2 of the Cochrane risk-of-bias tool for randomized trials (Figure 1) [21]. The risk-of-bias tool assesses the following domains: bias arising from the randomization process, bias due to deviations from intended interventions, bias due to missing outcome data, bias in the measurement of the outcome, and bias in the selection of the reported results. Each bias was assigned 1 of 3 levels: low risk of bias, some concerns, or high risk of bias. The risk of bias of each studies was presented in traffic light plots.

### Analysis

We assessed heterogeneity using the *I*^2^ statistic. We interpreted the *I*^2^ value as in the Cochrane Handbook for Systematic Review of Interventions (0%-40% might not be important, 30%-60% may represent moderate heterogeneity, 50%-90% may represent substantial heterogeneity, and 75%-100% may represent considerable heterogeneity). The source was investigated if significant heterogeneity was observed. Publication bias was evaluated by visual inspection of the funnel plot if at least 10 studies were included in the analysis. We calculated a pooled standard deviation for studies where standard deviations were not reported [22]. The results were compared using a sensitivity analysis with or without studies of imputed standard deviations and study targeted to children and adolescent. All analyses were conducted with Review Manager (version 5.4, The Cochrane Collaboration) software.

We performed the following subgroup analyses:

- By type of psychotherapy included in the intervention (with or without ERP), as a systematic review showed the efficacy of ERP against OCD [14]
- By intervention devices, as we hypothesized that device characteristics would influence the results. We planned to include portability with mobile phone, interaction with computer, and gamification. Portability may make it easy for participants to conduct ERP. Interaction and gamification may motivate participants to continue the intervention. However, no study included in this review used a mobile phone or gamification. As a result, we conducted a subgroup analysis with and without interaction with the system and intervention using a treatment manual via email or computer display. Interaction with the system means that participants can automatically get responses from a computer system without human contact
- By study duration or number of sessions to examine the influence of duration. We conducted an analysis on studies with a duration equal to or less than 4 weeks and studies over 4 weeks, as the median and mode of the included study duration was 4 weeks. We could not conduct subgroup analysis by session because no studies reported the number of sessions conducted
- By type of control arm, conducted as post hoc analysis, as a recent study showed that effect size may differ according to the control condition [23]. The subgroup differences were interpreted as suggestive when *P*<.10, in consideration of the small number of included studies and difficulty finding subgroup interactions.

The quality of evidence for primary outcomes was evaluated according to the GRADE (Grading of Recommendations, Assessment, Development, and Evaluations) rating [24].

## Results

### Search Results

**Figure 2:**
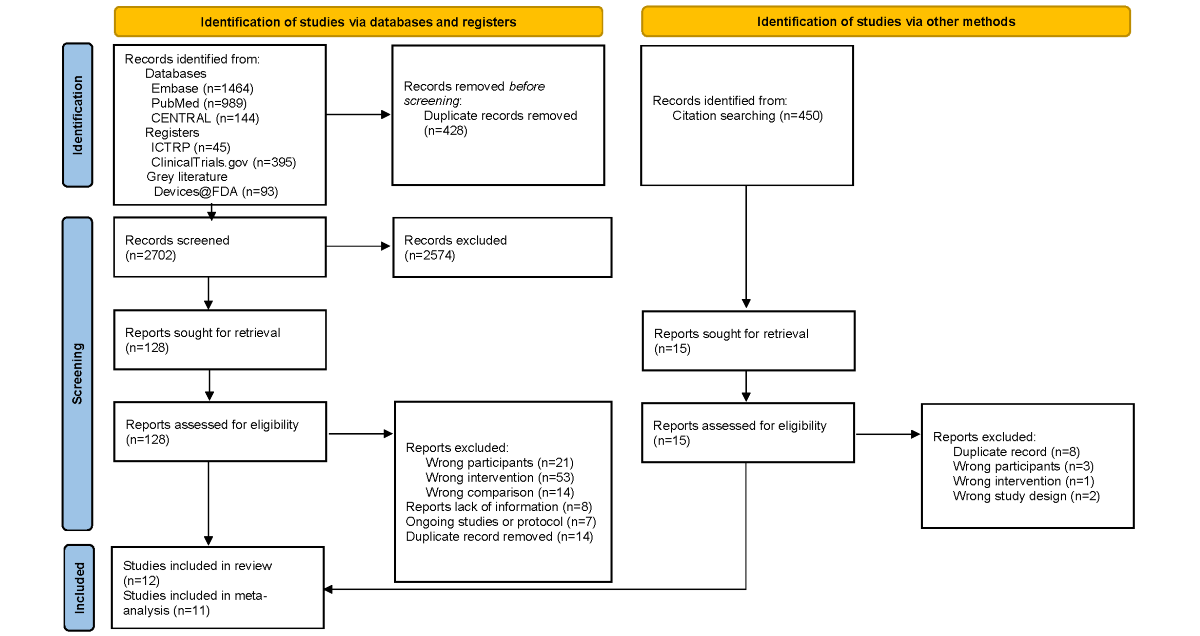
Preferred Reporting Items for Systematic Reviews and Meta-Analyses flow chart. CENTRAL: Cochrane Central Register of Controlled Trials; ICTRP: International Clinical Trials Registry Platform.

We identified 3130 references and excluded 2574 studies after assessing the title and abstracts. We retrieved 128 full-text papers, excluded 117 studies, and included 11 studies. We inspected the citations of the 11 studies and found 1 study to include. Finally, a total of 12 studies were included in the review, but we could not obtain additional data from the author of 1 study [25]. As a result, 11 studies with a total of 983 participants were included in the meta-analysis (Figure 2).

### Characteristics of Included Studies

As seen in Table 1, all included studies were parallel group, individually RCTs. One was a 3-armed study [26], while others were 2-armed. The mean sample size per arm was 45 (range 9-100).

Participants were recruited in European countries in 3 studies [27-29], in North America in 2 studies [26,30], and cross-continental in 1 study [31], but recruitment method was unclear in the other studies because it was done through the internet [32-36]. Diagnosis was based on DSM-IV-TR in 2 studies [27,28], DSM-IV in 1 study [26], health professional diagnosis using unclear diagnostic criteria in 5 studies [32-34,36,37], and the OCD symptom scale in 3 studies [29,30,38]. The proportion of women ranged from 42% to 83%. The mean age in a study targeting adolescents was 15 years [27], while others ranged from 28 to 41 years.

Interventions included computer-assisted cognitive training [30], behavioral therapy [26], metacognitive training [28,33,36], association splitting [39], inference-based therapy [37], competitive memory training [32], and CBT [29,38]. Of the included studies, Moritz et al [36] conducted 7 of them; however, only 3 used the same or a revised version of the intervention among them [28,33]. As for the component of therapy, 6 studies included exposure therapy [26,27,29,32,36,38]. ERP was used in 5 studies [26,27,29,36,38], and interoceptive exposure was used in 1 study [32]. Two studies explicitly examined the interaction between the system and the participants [26,29]. Three studies used a computer display that presented text-based online slides [38], text, video, audio elements, photos, illustrations [29], and a scenario with missing words that patients filled in [27]. These studies did not use gamification and did not include mobile devices. No studies used combination therapy; all but 1 study [30] allowed adjunctive medication.

**Table 1:** Characteristics of included studies.

Author; year; citation; country; study design	Participants		Interventions	Outcomes
	Diagnosis; sex; medications	Age (years), mean (SD); baseline severity, mean (SD)		
Calkins et al [30]; North America; RCT^a^	Dx: OCI-R^b^>15; Sex: CCT^c^ arm: 54.2% women; PVT^d^ arm 62.5%; meds^e^: unclear	Age: CCT arm 27.9 (SD 14.1); PVT arm 30 (SD 13.8); Severity: OCI-R CCT arm 28.9 (SD 11.1); PVT arm 30.8 (SD 0.9)	CCT n=24; duration 2 weeks; exposure: no; cognitive modification: no; device: computer; interaction: no; gamification: no but a kind of task; PVT n=24; duration 2 weeks	OCI-R; BDI-II^f^; PANAS^g^; PSWQ^h^
Greist et al [26]; North America; RCT	Dx: DSM-IV^i^; Sex: 42% women; meds: yes	Age: 39 (SD 12); Severity (Y-BOCS^j^): BT STEPS^k^ arm 24.6 (SD 4.3); systematic relaxation arm 25.8 (SD 5.1)	BT STEPS n=74; duration 10 weeks; exposure: yes (ERP^l^); cognitive modification: unclear; device: computer-driven interactive voice response system and workbook; interaction: yes; gamification: no; Systematic relaxation n=75; duration 10 weeks; Clinician-guided behavior therapy	Y-BOCS; PGI-I^m^; CGI scale^n^; WSAS^o^; HAM-D^p^; SRI^q^ medication status; treatment expectations; treatment satisfaction
Haushildt et al 2016 [28]; Europe; RCT	Dx: DSM-IV; Sex: myMCT arm: 67.2% women; psychoeducation arm: 67.2%; Meds: yes	Age: myMCT arm 38.41 (SD 11.61); psychoeducation arm 39.64 (SD 9.88); Severity (Y-BOCS): myMCT arm 22.56 (SD 6.58); psychoeducation arm 21.45 (SD 6.42)	myMCT n=64; duration 4 weeks; Device: pdf file through email; exposure: no; cognitive modification: yes (metacognitive training, association splitting); interaction: no; gamification: no; Psychoeducation n=64; duration 4 weeks	Y-BOCS, BDI^r^, OBQ^s^
Moritz et al 2010 [33]; recruited from internet forums; RCT	Dx: OCD^t^ diagnosis made by health care professionals; Sex: myMCT arm: 62.8% women; waiting list arm: 72.1%; Meds: yes	Age: myMCT arm 34.95 (SD 11.87); waiting list arm 34.09 (SD 9.41); Severity (Y-BOCS): myMCT arm 18.6 (SD 6.86); waiting list arm 19.98 (SD 5.9)	myMCT n=43; duration 4 weeks; device: pdf file through email; exposure: no; cognitive modification: yes (metacognitive training); Waiting list n=43; duration 4 weeks.	Y-BOCS, OCI-R, BDI-SF^u^
Moritz & Jelinek 2011 [34]; recruited from internet forums; RCT	Dx: OCD diagnosis made by health care professionals; Sex: AS^v^ arm: 56.5% women; waiting list arm: 78.3%; Meds: yes	Age: AS arm 36.0 (SD 9.81); waiting list arm 36.3 (SD 9.66); Severity (Y-BOCS): AS arm 21.96 (SD 8.17); waiting list arm 22.83 (SD 6.66)	AS n=43; duration 4 weeks; exposure: no; cognitive modification: yes (association splitting); device: treatment manual through email; interaction: no; gamification: no; Waiting list n=43; duration 4 weeks	Y-BOCS, OCI-R, BDI
Moritz et al 2015 [37]; English-speaking self-help groups and institutions devoted to research and treatment of OCD; RCT	Dx: externally verified diagnosis of OCD; Sex: IBT^w^ arm: 64% women; waiting list arm: 60%; Meds: yes	Age: IBT arm 36.88 (SD 13.14); waiting list arm 34.32 (SD 10.79); Severity (Y-BOCS): IBT arm 22.64 (SD 7.56); waiting list arm 21.48 (SD 7.38)	IBT n=25; duration 4 weeks; exposure: no; cognitive modification: yes (association splitting); device: treatment manual through email; interaction: no; gamification: no; Waiting list n=25; duration 4 weeks	Y-BOCS, OCI-R, ICQ^x^, WHOQOL-BREF^y^
Moritz et al 2018 [36]; online forum on OCD, Facebook OCD group, Yahoo newsgroups devoted to OCD; RCT	Dx: diagnosis by a mental health specialist; Sex: myMCT arm: 71.4% women; waiting list arm: 82.9%; Meds: yes	Age: myMCT arm 38.17 (SD 11.96); waiting list arm 39.34 (SD 14.52); Severity (Y-BOCS): myMCT arm 23.09 (SD 5.93); waiting list arm 21.74 (SD 6.23)	myMCT n=36; duration 6 weeks; exposure: yes (ERP); cognitive modification: yes; other: mindfulness; device: treatment manual through email; interaction: no; gamification: no; Waiting list n=36; duration 6 weeks	Y-BOCS, OCI-R, PHQ-9^z^, Maladaptive and Adaptive Coping Scale, PSQ*^aa^
Salemink et al 2015 [27]; Europe; RCT	Dx: DSM-IV-TR ^bb^; Sex: CBM-I^cc^ arm: 55.6% women; psychological placebo arm: 71.4%; Meds: yes	Age: CBM-I arm 15.6 (SD 2.4); psychological placebo arm 9 (SD 15.1); Severity (Children’s Y-BOCS): CBM-I arm 23.9 (SD 7.6); psychological placebo arm, 20.4 (SD 4.3)	CBM-I n=12; duration 1.6 weeks; exposure: yes (interoceptive exposure); cognitive modification: yes (cognitive bias modification training); device: computer; interaction: yes; gamification: no; Waiting list n=9; duration 1.6 weeks	Y-BOCS, OBQ-CV^dd^, RCADS^ee^, CDI^ff^
Schneider et al 2015 [32]; recruited from self-help forum through internet; RCT	Dx: diagnosis by a health care professional; Sex: COMET^gg^ arm: 55.9% women; waiting list arm: 61.3%; Meds: yes	Age: COMET arm 37.47 (SD 10); psychological placebo arm 37.06 (SD 10.3); Severity (Y-BOCS): COMET arm 18.5 (SD 5.95); waiting list arm 19.84 (SD 5.99)	COMET n=34; duration 4 weeks; exposure: yes (interoceptive exposure); cognitive modification: yes (competitive memory training); device: pdf manual through email; interaction: no; gamification: no; Waiting list n=34; duration 4 weeks	Y-BOCS, OCI-R, BDI-SF, RSES^hh^
Schröder et al 2020 [29]; Europe; RCT	Dx: Y-BOCS >7; Sex: iCBT^ii^ arm: 75% women; CAU^jj^ arm: 78.13%; Meds: yes	Age: iCBT arm 41.45 (SD 12.15); CAU arm 38.98 (SD 11.55); Severity (Y-BOCS): iCBT arm 20.2 (SD 6.29); CAU arm 20.17 (SD 5.73)	iCBT n=64; duration 8 weeks; exposure: yes (ERP); cognitive modification: yes (metacognitive training); other, mindfulness; device: computer (text, video, audio, photo, illustration); interaction: yes; gamification: no; CAU n=64; duration 8 weeks	Y-BOCS, OCI-R, OBQ-44^kk^, WHOQOL-BREF
Wootton et al 2019 [38]; cross-continental; RCT	Dx: Y-BOCS ≥14; Sex: ICBT^kk^ arm: 81.5% women; waiting list arm: 81.3%; Meds: yes	Age: ICBT arm 34.03 (SD 10.8); waiting list arm 33.39 (SD 10.25); Severity (Y-BOCS): ICBT arm 22.52 (SD 4.91); waiting list arm 22.44 (SD 5.55)	ICBT n=90; duration 8 weeks; exposure: yes (ERP); cognitive modification: no; device: text-based online slides; interaction: no; gamification: no; Waiting list n=100; duration 8 weeks	Y-BOCS, DOCS^ll^, PHQ-9

^a^RCT: randomized controlled trial.

^b^OCI-R: Obsessive-Compulsive Inventory–Revised.

^c^CCT: computerized cognitive control.

^d^PVT: peripheral vision training.

^e^meds: adjunctive medications.

^f^BDI-II: Beck Depression Inventory–Second Edition.

^g^PANAS: Positive and Negative Affectivity Scale.

^h^PSWQ: Penn State Worry Questionnaire.

^i^DSM-IV: Diagnostic and Statistical Manual of Mental Disorders, Fourth Edition.

^j^Y-BOCS: Yale-Brown Obsessive-Compulsive Scale.

^k^BT STEPS: Behavior Therapy Self-Help System.

^l^ERP: exposure and response prevention.

^m^PGI-I: Patient Global Impression of Improvement.

^n^CGI scale: Clinical Global Impression scale.

^o^WSAS: Work and Social Adjustment Scale.

^p^HAM-D: Hamilton Depression Rating Scale.

^q^SRI: serotonin reuptake inhibitor.

^r^BDI: Beck Depression Inventory.

^s^OBQ: Obsessive Belief Questionnaire.

^t^OCD: obsessive-compulsive disorder.

^u^BDI-SF: Beck Depression Inventory–Short Form.

^v^AS: association splitting.

^w^IBT: inference-based therapy.

^x^ICQ: Inferential Confusion Questionnaire.

^y^WHOQOL-BREF: Brief Quality of Life Questionnaire of the World Health Organization.

^z^PHQ-9: Patient Health Questionnaire.

^aa^PSQ: Patient Satisfaction Questionnaire.

DSM-IV-TR: Diagnostic and Statistical Manual of Mental Disorders, Fourth Edition, Text Revision

^cc^CBM-I: Cognitive Bias Modification of Interpretation training.

^dd^OBQ-CV: Obsessive Belief Questionnaire–Child Version.

^ee^RCADS: Revised Child Anxiety and Depression Scale.

^ff^CDI: Children’s Depression Inventory.

^gg^COMET: Competitive Memory Training.

^hh^RSES: Rosenberg Self-Esteem Scale.

^ii^iCBT: internet-based cognitive-behavioral therapy.

^jj^CAU: care-as-usual.

^kk^OBQ-44: Obsessive Belief Questionnaire–44 item.

^ll^DOCS: Dimensional Obsessive-Compulsive Scale.

### Bias Arising From Randomization Process

Most of the studies did not provide information on allocation sequence concealment. Of those who provided details, Schneider et al [32] used an online randomization and allocation system, Salemink et al [27] suspected baseline imbalance, and Wootton et al [38] did not blind allocation to the clinician assessing participants.

### Bias Due to Deviations From Intended Interventions

Six studies used waiting lists [25-27,29-31], and the other 5 used psychological placebo or treatment as control arms [19-23]. The percentage of dropouts was unbalanced between the arms and probably affected the results except for 2 studies, where the authors conducted analyses to confirm the deviations did not affect the outcome [33,38].

### Bias Due to Missing Outcome Data

Most of the studies were missing more than 5% of the data, were unbalanced, and neglected to provide reasons for dropouts [19-22,25-27,29,30]. One study, however, had no missing data [30], and another conducted an analysis to prove that missingness did not affect the true value [38].

### Bias in Measurement of Outcome

Primary efficacy outcome was measured by the self-rated Y-BOCS. It was unclear if knowledge of the intervention influenced the results.

### Bias in Selection of Reported Results

We found the protocol for the RCT by Schröder et al [29] but no others; therefore, selection of the reported results was unclear.

### Primary Outcomes

#### Short-term Subjective Improvement of OCD Symptoms

**Figure 3:**
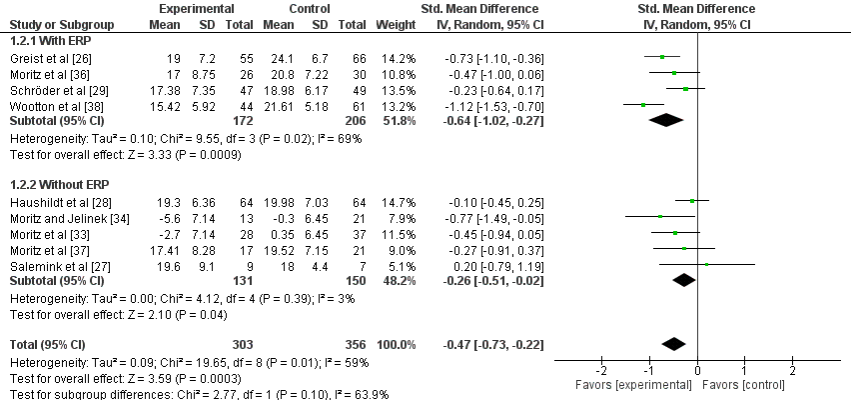
Forest plot of short-term improvement of obsessive-compulsive disorder symptoms. ERP: exposure and response prevention.

Unguided computer-assisted self-help therapy was more effective than the waiting list and psychological placebo in terms of short-term subjective improvement of OCD symptoms (SMD −0.47, 95% CI −0.73 to −0.22; 9 studies; 659 participants). There was moderate heterogeneity (*I*^2^=59%; Tau^2^=0.09; Figure 3). The quality of evidence was very low due to the risk of bias of the included studies and inconsistency of the results.

Heterogeneity decreased to 24% after we excluded a study that reported a large number of dropouts before the intervention began [38]. This may have caused participants with high motivation to start the intervention, exaggerating the therapeutic effect. The improvement in OCD symptoms in the intervention arms remained larger than that in the control arms after exclusion of the study (SMD −0.38, 95% CI −0.58 to −0.18; 8 studies; 554 participants).

We imputed standard deviations in 2 studies [33,34]. The exclusion of these studies did not substantially change the result (SMD −0.44, 95% CI −0.76 to −0.12; 7 studies; 560 participants). The sensitivity analysis without studies targeted to child and adolescent [27] did not substantially change the result (SMD –0.51; 95% CI, –0.56 to –0.04; 8 studies; 643 participants; *I*^2^=61%).

The subgroup analysis limited to those with ERP tended to strengthen the efficacy of unguided computer-assisted self-help therapy (SMD −0.64, 95% CI −1.02 to −0.27; 4 studies; 378 participants; *I*^2^=69%) [26,29,36,38,40] compared with those without ERP (SMD −0.26, 95% CI −0.51 to −0.02; 5 studies; 281 participants; *I*^2^=3%) [27,28,33,34,37]. The test for subgroup difference suggested a subgroup difference (*P*=.10).

The majority of the studies only sent the treatment manual via email. The subgroup analysis limited to those studies with treatment manual showed that the intervention was more effective than the control arm (SMD −0.44, 95% CI −0.68 to −0.20; 6 studies; 442 participants; *I*^2^=66%) [26,28,33,34,36,37]. In comparison, the analysis limited to those using computer display showed no significant difference between the intervention and control groups (SMD −0.46, 95% CI −1.21 to 0.29; 3 studies; 217 participants; *I*^2^=83%; Multimedia Appendix 3) [27,29,38]. The test for subgroup difference showed no significant subgroup difference (*P*=.96). This tendency became more evident after excluding a study where dropouts were relatively large before the interventions started (SMD −0.17, 95% CI −0.54 to 0.20; 2 studies; 112 participants; *I*^2^=0%) [27,29]. However, the test for subgroup difference still showed no significant subgroup difference (*P*=.24).

The subgroup analysis limited to studies with some kind of interaction with systems showed no significant difference between the intervention and control groups (SMD −0.38, 95% CI –0.84 to 0.09; 3 studies; 233 participants; *I*^2^=60%) [26,27,29], whereas an analysis limited to studies without interaction showed the intervention was more effective than the control arm treatment (SMD −0.52, 95% CI −0.87 to −0.17; 6 studies; 426 participants; *I*^2^=55%). The test for subgroup difference showed no significant subgroup difference (*P*=.63; Multimedia Appendix 4).

In terms of duration of the intervention, studies with 4 weeks or less of intervention showed no significant difference between the intervention and control groups (SMD −0.20, 95% CI −0.45 to 0.06; 4 studies; 247 participants; *I*^2^=0%) [27,28,33,37], whereas those with a duration of over 4 weeks showed that interventions were more effective than control (SMD −0.64, 95% CI −1.02 to −0.27; 4 studies; 378 participants; *I*^2^=69%; Multimedia Appendix 5) [26,29,36,38]. The test for subgroup difference suggested a subgroup difference (*P*=.05). The number of sessions conducted was unclear, as they were self-help interventions.

The subgroup analysis limited to studies with waiting list as control arm showed that the intervention was significantly more effective than the waiting list (SMD −0.56, 95% CI −0.91 to −0.22; 6 studies; 314 participants; *I*^2^=51%) [27,33,34,36,38], whereas studies with psychological placebo as control arm showed no significant difference between the intervention and control groups (SMD −0.35, 95% CI −0.74 to 0.03; 3 studies; 345 participants; *I*^2^=69%). The test for subgroup difference showed no significant subgroup difference (*P*=.43; Multimedia Appendix 6).

#### Dropout for Any Reason at Posttreatment

**Figure 4:**
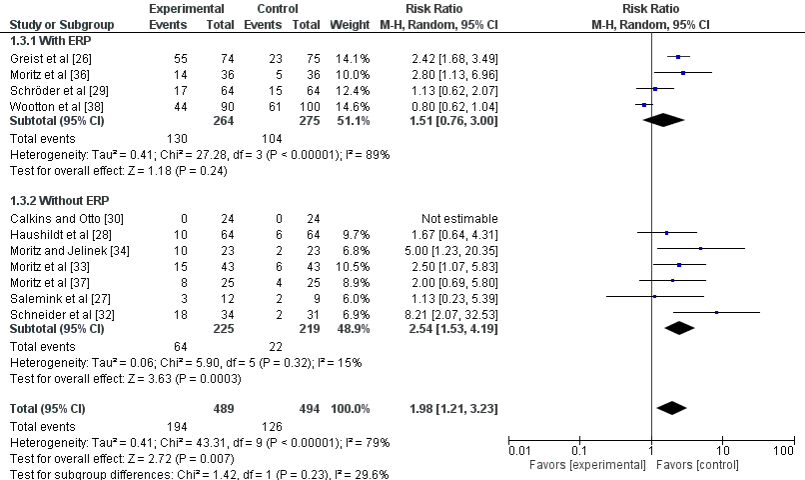
Forest plot of dropout for any reason at posttreatment.

**Figure 5:**
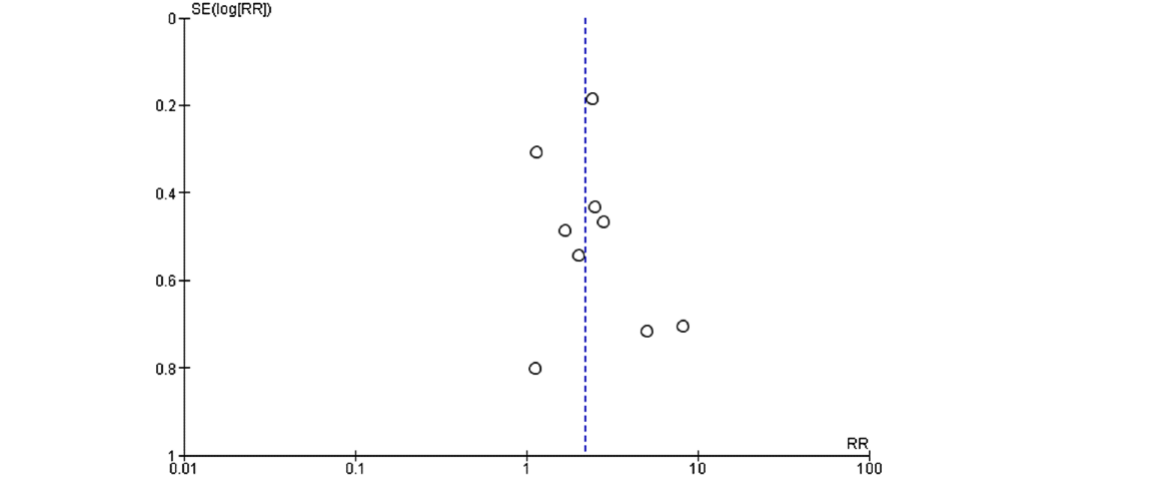
Funnel plot to assess for publication bias of dropout for any reason.

Unguided computer-assisted self-help therapy had more dropouts for any reason than waiting list or psychological placebo (risk ratio [RR] 1.98, 95% CI 1.21 to 3.23; 11 studies, 983 participants; Figure 4). The visual inspection of the funnel plot suggested publication bias (Figure 5). In fact, there was considerable heterogeneity (*I*^2^=79%, Tau^2^=0.41). The quality of evidence was very low due to the risk of bias, inconsistency of results, and suspected publication bias.

Heterogeneity decreased to 29% after excluding a study that had also been excluded from the sensitivity analysis of the short-term improvement of OCD symptoms [38]. The dropouts for any reason in the intervention arm were still larger than those in the control arms after exclusion of the study (RR 2.19, 95% CI 1.56 to 3.07; 11 studies; 793 participants).

The sensitivity analysis without studies targeted to child and adolescent [27] did not substantially change the result (RR 2.06, 95% CI 1.23 to 3.45; 10 studies; 962 participants; *I*^2^=82%).

The subgroup analysis limited to those with ERP showed no significant difference between the intervention and control arms in dropout for any reason (RR 1.51, 95% CI 0.76 to 3.00; 4 studies; 539 participants; *I*^2^=89%) [26,29,36,38]. Among these 4 studies, the 2 with manual-based treatment had significantly more dropouts in the intervention arm than in the control arm (RR 2.47, 95% CI 1.76 to 3.47; 221 participants; *I*^2^=0%) [26,36], and the 2 with online slide or video, audio, photo, and illustration were not significantly different from the control arm in dropout for any reason (RR 0.86, 95% CI 0.65 to 1.13; 4 studies; 318 participants; *I*^2^=10%) [29,38]. The studies with manual-based treatment [26,36] had intervention durations of 10 weeks and 6 weeks, respectively; the online interventions [29,38] were both 8 weeks in duration. The analysis limited to those without ERP showed that the intervention arm had significantly more dropout for any reason than the control arm (RR 2.54, 95% CI 1.53 to 4.19; 7 studies; 444 participants; *I*^2^=15%) [27,28,30,32-34,37].

The subgroup analysis limited to those using computer display showed no significant difference between the intervention and control group with respect to dropout for any reason (RR 0.85, 95% CI 0.67 to 1.08; 3 studies; 339 participants; *I*^2^=0%) [27,29,38]. The analysis of studies using a treatment manual showed that the intervention had significantly more dropout for any reason than the control arm (RR 2.55, 95% CI 1.93 to 3.36; 8 studies; 644 participants; *I*^2^=0%; Multimedia Appendix 7) [26,28,30,32-34,36,37]. The test for subgroup difference suggested a subgroup difference (*P*=.05).

The subgroup analysis limited to studies with some kind of interaction with systems showed that the intervention arm had a significantly higher dropout for any reason than the control (RR 1.65, 95% CI 0.89 to 3.06; 3 studies; 233 participants; *I*^2^=60%) [26,27,29]. This trend was the same as the analysis of those without interaction (RR 2.33, 95% CI 1.11 to 4.88; 8 studies; 685 participants; *I*^2^=82%; Multimedia Appendix 8). The test for subgroup difference showed no significant subgroup difference (*P*=.48). There was substantial heterogeneity in both analyses.

In terms of duration of the intervention, studies with interventions of 4 weeks or less had significantly more dropouts than the control arm (RR 2.54, 95% CI 1.53 to 4.19; 7 studies; 444 participants; *I*^2^=15%), whereas those with more than 4 weeks of intervention showed no significant difference in dropout for any reason between the intervention and control arms (RR 1.51, 95% CI 0.76 to 3.00; 4 studies; 539 participants; *I*^2^=89%; Multimedia Appendix 9). The test for subgroup difference showed no significant subgroup difference (*P*=.23), The latter analysis included the same studies as the analysis of studies with ERP.

The subgroup analysis by control arm showed that the intervention group had significantly more dropouts for any reason than control groups (waiting list control RR 1.79, 95% CI 1.24 to 2.58; 7 studies; 530 participants; *I*^2^=78%; others RR 2.76, 95% CI 1.73 to 4.38; 4 studies; 453 participants; *I*^2^=81%; Multimedia Appendix 10).

### Secondary Outcomes

#### Short-term Response Rate

The unguided computer-assisted self-help therapy had a more short-term response than the waiting list/psychological placebo (RR 1.93, 95% CI 1.16 to 3.21; 2 studies; 249 participants). Heterogeneity was negligible (*I*^2^=18%, Tau^2^=0.02).

#### Short-term Improvement of Anxiety

One study evaluated short-term improvements in anxiety [27]. There was no significant difference between the unguided computer-assisted self-help therapy and waiting list/psychological placebo in the improvement of anxiety (mean difference [MD] −6.20, 95% CI −20.38 to 7.98; 1 study; 16 participants).

#### Short-term Improvement of Depression

The improvement in depression was significantly greater in unguided computer-assisted self-help therapy than in the waiting list/psychological placebo (SMD −0.19, 95% CI −0.35 to −0.02; 7 studies; 560 participants). Heterogeneity was negligible (*I*^2^=0%, Tau^2^=0). The sensitivity analysis without studies targeted to child and adolescent [27] did not substantially change the result (SMD –0.18, 95% CI –0.35 to –0.01; 6 studies; 544 participants; *I*^2^=0%).

#### Quality of Life

There was no significant difference in short-term improvement of QOL between the unguided computer-assisted self-help therapy and waiting list/psychological placebo (MD 0.48, 95% CI −4.06 to 5.03; 2 studies; 134 participants). Heterogeneity was negligible (*I*^2^=0%, Tau^2^=0).

#### Other Outcomes

No study has evaluated outcomes longer than 6 months. One study evaluated the Y-BOCS and Beck Depression Inventory–Second Edition (BDI-II) at 6 months [28]. There was no difference in the improvement of Y-BOCS (MD 0.46, 95% CI −2.02 to 2.94; 128 participants) and BDI-II (MD 0.47, 95% CI −2.65 to 3.59; 128 participants) at 6 months between unguided computer-assisted self-help therapy and waiting list/psychological placebo.

## Discussion

### Summary of Main Outcomes

We included 11 studies with a total of 983 participants. The results indicated that unguided computer-assisted self-help therapy was moderately more effective than waiting lists or a attention placebo, which was confirmed by sensitivity analyses. In addition, there were no significant differences in acceptability as measured by dropout for any reason between the 2 arms.

Subgroup analysis limited to studies with ERP or interventions of 4 weeks or less tended to strengthen the efficacy of unguided computer-assisted self-help therapy, although the number of included studies in these analyses was small. Moreover, there was no significant difference in efficacy between the 2 groups when the analysis was limited to studies using computer display or studies with the interaction between participants and systems.

For the acceptability measured by dropout for any reason, subgroup analysis limited to studies with ERP did not change the result, but the intervention arm had more dropouts when the analysis was limited to studies using treatment manual via email. In terms of intervention duration, analysis limited to studies of 4 weeks or less showed that the intervention arm had a greater number of dropouts than the control arm.

Short-term responses for secondary outcomes supported the efficacy of unguided computer-assisted self-help therapy; however, only 2 studies were included in the analysis. The short-term improvement of depression was greater with unguided computer-assisted therapy, but 2 studies reported no significant difference in the improvement of QOL and 1 study reported no difference in level of anxiety. There are no studies with long-term outcomes.

### Comparison With Other Systematic Reviews and Strengths of This Review

There were 3 systematic reviews and meta-analyses related with this study. All results favored the interventions. Firth et al [12] indicated a small-to-moderate effect (Hedges *g*=0.325) of a smartphone intervention on the total symptoms of anxiety in comparison with control conditions, which did not exclude face-to-face support. Tumur et al [10] showed that the effect size of Y-BOCS in a computer-assisted CBT intervention named BT Step was 0.84, which was the only intervention included in the analysis. The study conducted by Peacy et al [11], which was most similar to this study, showed that the effect size of self-administered self-help intervention was small (Hedges *g*=0.33).

In accordance with previous reviews, our review favored unguided computer-assisted self-help therapy against control arms, and the effect size was moderate (SMD −0.47). Although Pearcy et al [11] showed a small effect size of the intervention, they included quasi-experimental studies, and the RCT conducted by Greist et al [26] was misclassified to predominantly self-help; the study author confirmed was a self-administered therapy upon our inquiry.

This review reveals the acceptability of self-guided computer-assisted therapy for OCD measured by dropout for any reason. Future systematic reviews on self-guided OCD therapy should include the analysis of acceptability as one of the problems of self-guided therapy [41,42].

### Importance of ERP and Comparison to Intervention With Human Contact

This study reconfirmed the importance of ERP in the treatment of OCD. The results of the meta-analysis showed that interventions with ERP were significantly more effective than those without ERP. However, human contact may strengthen the effect of ERP. The past meta-analyses on intervention with ERP compared with control condition showed that the SMDs of obsessive-compulsive symptoms were 1.16 and 0.74, respectively [14,15]. The former did not include computer-assisted interventions and the latter did. Our results showed that the effect of unguided computer-assisted self-help interventions without human contact expressed as SMD was 0.64. These facts suggest the importance of human contact in ERP. In fact, one of the meta-analyses listed above showed that the SMD of therapist-controlled ERP (SMD 1.58) was greater than that of self-controlled ERP (SMD 0.81) [15]. Unguided computer-assisted self-help interventions without human contact should include ERP, and future studies should examine what factors of human contact strengthen the effect of ERP.

### Duration of Intervention and Its Influence on Effect and Dropouts

Our results showed that interventions with a duration over 4 weeks were more effective and tended to have fewer dropouts than interventions of 4 weeks or less. Avoiding interventions shorter than 4 weeks is recommended, considering the negligible heterogeneity of the results. However, it is unclear how long the intervention should be.

Several studies indicated that increment of treatment effect would decrease as the number of sessions increases [16,43], and a study suggested that patients tend to end therapy when they are satisfied with their improvement [17]. An intervention with a flexible number of sessions may be one option to determine the optimal number of sessions.

### Comparison With Other Apps

One systematic review showed that highly rated anxiety apps contain gamification (32%) and social elements including chat and communication with others (46%) [44]. The studies included in our systematic review did not use gamification or mobile devices, and only 2 studies used interaction. Future studies of self-guided computer-assisted therapy for OCD should include these elements to increase efficacy and acceptability.

### Limitations

This study has several limitations. First, this study did not include active interventions as a comparison. While this would increase the number of included studies and precision, such an analysis may lead to an underestimation of the target intervention’s efficacy. Second, the overall risk of bias of the included studies was high, which led to downgrading the quality of evidence. However, this was unavoidable since a waiting list was the comparison arm, and the primary efficacy outcome was measured using a self-administered questionnaire. Future studies should use a psychological placebo to keep participants blinded to the intervention and the objective outcomes. Third, we did not consider sponsorship bias, which may favor the results of the intervention. However, as it seems that all authors developed the intervention, the results of this review may have overestimated the effect. The test of sponsorship bias should be initiated at the study design level. Fourth, the heterogeneity of results was moderate to considerable. This suggests that various factors are related to the effect of the computer-assisted self-help interventions in patients with OCD, such as the module, duration, modality of presenting intervention, gamification, and intervention. The number of studies on the computer-assisted self-help interventions in patients with OCD is still small, as shown in this study. More studies to explore and optimize the effect of the intervention should be conducted.

### Conclusions

Our study suggests that unguided computer-assisted self-help therapy for OCD is effective compared to waiting lists or psychological placebo. An ERP component and intervention duration of more than 4 weeks may strengthen the efficacy without worsening the acceptability of the therapy.

However, the included studies did not effectively use the merits of computerization. Few studies have examined the interaction between participants and systems, and none of the studies used gamification. Furthermore, most studies only used text-based interventions. No study used a mobile device. Portability seems to be useful for intervention components, such as self-monitoring and in vivo exposure; therefore, future studies should examine these factors.

## Appendix

Multimedia Appendix 1 PRISMA checklist.

Multimedia Appendix 2 Search strategy.

Multimedia Appendix 3 Forest plot of short-term improvement of obsessive-compulsive disorder symptoms: subgroup analysis by treatment type.

Multimedia Appendix 4 Forest plot of short-term improvement of obsessive-compulsive disorder symptoms: subgroup analysis with and without interaction.

Multimedia Appendix 5 Forest plot of short-term improvement of obsessive-compulsive disorder symptoms: subgroup analysis by treatment duration.

Multimedia Appendix 6 Forest plot of short-term improvement of obsessive-compulsive disorder symptoms: subgroup analysis by control condition.

Multimedia Appendix 7 Forest plot of dropout for any reason at posttreatment: subgroup analysis by treatment type.

Multimedia Appendix 8 Forest plot of dropout for any reason at posttreatment: subgroup analysis with or without interaction.

Multimedia Appendix 9 Forest plot of dropout for any reason at posttreatment: subgroup analysis by treatment duration.

Multimedia Appendix 10 Forest plot of dropout for any reason at posttreatment: subgroup analysis by control condition.

## Abbreviations

BDI-II: Beck Depression Inventory–Second Edition
BT STEPS: Behavior Therapy Self-Help System
CBT: cognitive behavioral therapy
DSM-5: Diagnostic and Statistical Manual of Mental Disorders, Fifth Edition
DSM-IV-TR: Diagnostic and Statistical Manual of Mental Disorders, Fourth Edition, Text Revision
ERP: exposure and response prevention
GRADE: Grading of Recommendations, Assessment, Development, and Evaluations
MD: mean difference
OCD: obsessive-compulsive disorder
OCI-R: Obsessive-Compulsive Inventory–Revised
PRISMA: Preferred Reporting Items for Systematic Reviews and Meta-Analyses
PROSPERO: International Prospective Register of Systematic Reviews
QOL: quality of life
RCT: randomized controlled trial
RR: risk ratio
SMD: standardized mean difference
Y-BOCS: Yale-Brown Obsessive-Compulsive Scale

## References

[1] American Psychiatric Association (2013). Diagnostic and Statistical Manual of Mental Disorders, 5th Edition.

[2] Eaton WW, Martins SS, Nestadt G, Bienvenu OJ, Clarke D, Alexandre P (2008). The burden of mental disorders. Epidemiol Rev.

[3] Skapinakis P, Caldwell DM, Hollingworth W, Bryden P, Fineberg NA, Salkovskis P, Welton NJ, Baxter H, Kessler D, Churchill R, Lewis G (2016). Pharmacological and psychotherapeutic interventions for management of obsessive-compulsive disorder in adults: a systematic review and network meta-analysis. Lancet Psychiatry.

[4] McHugh RK, Whitton SW, Peckham AD, Welge JA, Otto MW (2013). Patient preference for psychological vs pharmacologic treatment of psychiatric disorders: a meta-analytic review. J Clin Psychiatry.

[5] (2005). Obsessive-compulsive disorder and body dysmorphic disorder: treatment. National Institute for Health and Care Excellence.

[6] Goodwin R, Koenen KC, Hellman F, Guardino M, Struening E (2002). Helpseeking and access to mental health treatment for obsessive-compulsive disorder. Acta Psychiatr Scand.

[7] García-Soriano G, Rufer M, Delsignore A, Weidt S (2014). Factors associated with non-treatment or delayed treatment seeking in OCD sufferers: a review of the literature. Psychiatry Res.

[8] Lind C, Boschen MJ, Morrissey S (2013). Technological advances in psychotherapy: implications for the assessment and treatment of obsessive compulsive disorder. J Anxiety Disord.

[9] Rogers MA, Lemmen K, Kramer R, Mann J, Chopra V (2017). Internet-delivered health interventions that work: systematic review of meta-analyses and evaluation of website availability. J Med Internet Res.

[10] Tumur I, Kaltenthaler E, Ferriter M, Beverley C, Parry G (2007). Computerised cognitive behaviour therapy for obsessive-compulsive disorder: a systematic review. Psychother Psychosom.

[11] Pearcy CP, Anderson RA, Egan SJ, Rees CS (2016). A systematic review and meta-analysis of self-help therapeutic interventions for obsessive-compulsive disorder: is therapeutic contact key to overall improvement?. J Behav Ther Exp Psychiatry.

[12] Firth J, Torous J, Nicholas J, Carney R, Rosenbaum S, Sarris J (2017). Can smartphone mental health interventions reduce symptoms of anxiety? A meta-analysis of randomized controlled trials. J Affect Disord.

[13] Ahmadvand A, Kavanagh D, Clark M, Drennan J, Nissen L (2019). Trends and visibility of "digital health" as a keyword in articles by JMIR Publications in the new millennium: bibliographic-bibliometric analysis. J Med Internet Res.

[14] Reid JE, Laws KR, Drummond L, Vismara M, Grancini B, Mpavaenda D, Fineberg NA (2021). Cognitive behavioural therapy with exposure and response prevention in the treatment of obsessive-compulsive disorder: a systematic review and meta-analysis of randomised controlled trials. Compr Psychiatry.

[15] Abramowitz JS (1996). Variants of exposure and response prevention in the treatment of obsessive-compulsive disorder: a meta-analysis. Behavior Therapy.

[16] Kopta SM (2003). The dose-effect relationship in psychotherapy: a defining achievement for Dr. Kenneth Howard. J Clin Psychol.

[17] Barkham M, Connell J, Stiles WB, Miles JNV, Margison F, Evans C, Mellor-Clark J (2006). Dose-effect relations and responsive regulation of treatment duration: the good enough level. J Consult Clin Psychol.

[18] Cuijpers P, Marks IM, van Straten A, Cavanagh K, Gega L, Andersson G (2009). Computer-aided psychotherapy for anxiety disorders: a meta-analytic review. Cogn Behav Ther.

[19] Edwards EA, Lumsden J, Rivas C, Steed L, Edwards LA, Thiyagarajan A, Sohanpal R, Caton H, Griffiths CJ, Munafò MR, Taylor S, Walton RT (2016). Gamification for health promotion: systematic review of behaviour change techniques in smartphone apps. BMJ Open.

[20] Page MJ, McKenzie JE, Bossuyt PM, Boutron I, Hoffmann TC, Mulrow CD, Shamseer L, Tetzlaff JM, Akl EA, Brennan SE, Chou R, Glanville J, Grimshaw JM, Hróbjartsson A, Lalu MM, Li T, Loder EW, Mayo-Wilson E, McDonald S, McGuinness LA, Stewart LA, Thomas J, Tricco AC, Welch VA, Whiting P, Moher D (2021). The PRISMA 2020 statement: an updated guideline for reporting systematic reviews. BMJ.

[21] Sterne JAC, Savović J, Page MJ, Elbers RG, Blencowe NS, Boutron I, Cates CJ, Cheng H, Corbett MS, Eldridge SM, Emberson JR, Hernán MA, Hopewell S, Hróbjartsson A, Junqueira DR, Jüni P, Kirkham JJ, Lasserson T, Li T, McAleenan A, Reeves BC, Shepperd S, Shrier I, Stewart LA, Tilling K, White IR, Whiting PF, Higgins JPT (2019). RoB 2: a revised tool for assessing risk of bias in randomised trials. BMJ.

[22] Furukawa TA, Barbui C, Cipriani A, Brambilla P, Watanabe N (2006). Imputing missing standard deviations in meta-analyses can provide accurate results. J Clin Epidemiol.

[23] Michopoulos I, Furukawa TA, Noma H, Kishimoto S, Onishi A, Ostinelli EG, Ciharova M, Miguel C, Karyotaki E, Cuijpers P (2021). Different control conditions can produce different effect estimates in psychotherapy trials for depression. J Clin Epidemiol.

[24] Guyatt GH, Oxman AD, Vist GE, Kunz R, Falck-Ytter Y, Alonso-Coello P, Schünemann HJ (2008). GRADE: an emerging consensus on rating quality of evidence and strength of recommendations. BMJ.

[25] Nieman D, Domen A, Kumar R, Harrison J, De Haan L, Denys D (2015). Cognitive remediation in psychiatric patients with an online cognitive game and assessment tool. Eur Neuropsychopharmacol.

[26] Greist JH, Marks IM, Baer L, Kobak KA, Wenzel KW, Hirsch MJ, Mantle JM, Clary CM (2002). Behavior therapy for obsessive-compulsive disorder guided by a computer or by a clinician compared with relaxation as a control. J Clin Psychiatry.

[27] Salemink E, Wolters L, de Haan E (2015). Augmentation of treatment as usual with online cognitive bias modification of interpretation training in adolescents with obsessive compulsive disorder: a pilot study. J Behav Ther Exp Psychiatry.

[28] Hauschildt M, Schröder J, Moritz S (2016). Randomized-controlled trial on a novel (meta-)cognitive self-help approach for obsessive-compulsive disorder (“myMCT”). J Obsessive Compuls Rel Dis.

[29] Schröder J, Werkle N, Cludius B, Jelinek L, Moritz S, Westermann S (2020). Unguided Internet-based cognitive-behavioral therapy for obsessive-compulsive disorder: a randomized controlled trial. Depress Anxiety.

[30] Calkins AW, Otto MW (2012). Testing the boundaries of computerized cognitive control training on symptoms of obsessive compulsive disorder. Cogn Ther Res.

[31] Wootton BM (2016). Remote cognitive-behavior therapy for obsessive-compulsive symptoms: a meta-analysis. Clin Psychol Rev.

[32] Schneider BC, Wittekind CE, Talhof A, Korrelboom K, Moritz S (2015). Competitive Memory Training (COMET) for OCD: a self-treatment approach to obsessions. Cogn Behav Ther.

[33] Moritz S, Jelinek L, Hauschildt M, Naber D (2010). How to treat the untreated: effectiveness of a self-help metacognitive training program (myMCT) for obsessive-compulsive disorder. Dialogues Clin Neurosci.

[34] Moritz S, Jelinek L (2011). Further evidence for the efficacy of association splitting as a self-help technique for reducing obsessive thoughts. Depress Anxiety.

[35] O'Connor KP, Aardema F, Bouthillier D, Fournier S, Guay S, Robillard S, Pélissier MC, Landry P, Todorov C, Tremblay M, Pitre D (2005). Evaluation of an inference-based approach to treating obsessive-compulsive disorder. Cogn Behav Ther.

[36] Moritz S, Hauschildt M, Murray SC, Pedersen A, Krausz M, Jelinek L (2018). New wine in an old bottle? Evaluation of myMCT as an integrative bibliotherapy for obsessive-compulsive disorder. J Obsessive Compulsive Rel Dis.

[37] Moritz S, Dietl C, Kersten JF, Aardema F, O'Connor K (2015). Evaluation of inference-based therapy (doubt therapy) as a self-help tool for obsessive-compulsive disorder. J Cogn Psychother.

[38] Wootton BM, Karin E, Titov N, Dear BF (2019). Self-guided internet-delivered cognitive behavior therapy (ICBT) for obsessive-compulsive symptoms: a randomized controlled trial. J Anxiety Disord.

[39] Moritz S, Russu R (2013). Further evidence for the efficacy of association splitting in obsessive-compulsive disorder: an internet study in a Russian-speaking sample. J Obsessive Compulsive Rel Dis.

[40] Wolters L, Salemink E, De Beek V, De Haan E (2015). Improving treatment: supplementing cognitive behavioral therapy with a cognitive bias modification training for children and adolescents with OCD. Eur Child Adolesc Psychiatry.

[41] Cuijpers P, Noma H, Karyotaki E, Cipriani A, Furukawa TA (2019). Effectiveness and acceptability of cognitive behavior therapy delivery formats in adults with depression: a network meta-analysis. JAMA Psychiatry.

[42] Yeung W, Chung K, Ho FY, Ho L (2015). Predictors of dropout from internet-based self-help cognitive behavioral therapy for insomnia. Behav Res Ther.

[43] Howard KI, Kopta SM, Krause MS, Orlinsky DE (1986). The dose-effect relationship in psychotherapy. Am Psychol.

[44] Drissi N, Ouhbi S, Janati Idrissi MA, Ghogho M (2020). An analysis on self-management and treatment-related functionality and characteristics of highly rated anxiety apps. Int J Med Inform.

