# Supplementary material for: Unguided Computer-Assisted Self-Help Interventions Without Human Contact in Patients With Obsessive-Compulsive Disorder: Systematic Review and Meta-analysis
Source: J Med Internet Res. 2022 Apr 21;24(4):e35940. doi: 10.2196/35940 (PMC9073609; doi:10.2196/35940)
Supplement: Multimedia Appendix 2 [file jmir_v24i4e35940_app2.docx]

Multimedia Appendix 1. Search strategy.

| Pubmed search conducted on 2021/7/28 n=989 |
| --- |
| #1 “Obsessive-Compulsive Disorder”[Mesh] or “OCD”[tiab] or “obsessive-compulsive”[tiab] or “obsessive　compulsive”[tiab] or obsess* or compulsi* or intrusi*  #2 “Self-Help Devices”[Mesh] or “Computer Communication Networks “[Mesh] or “Cell Phone”[Mesh] or “Cell Phone Use”[Mesh] or “telemedicine”[Mesh] or “Microcomputers “[Mesh] or mHealth[tiab] or telehealth[tiab] or telemedicine[tiab] or eHealth[tiab] or m-health[tiab] or e-health[tiab] or telecommunication*[tiab] or teleconference*[tiab] or cellular phone*[tiab] or cellular telephone*[tiab] or cell phone*[tiab] or mobile phone*[tiab] or mobile telephone*[tiab] or app[tiab] or apps[tiab] or application*[tiab] or mobile*[tiab] or smart*[tiab] or wireless*[tiab] or unguid*[tiab] or self-direct[tiab] or self-help[tiab] self-manag*[tiab] or self-act*[tiab] or selfadmin[tiab] or e-self-help[tiab] or self-guid*[tiab] or web[tiab] or internet[tiab] or online[tiab] or webs*****[tiab] or computer[tiab] or eTherapy[tiab] or e-therap*[tiab]  #3 "clinical study"[Publication Type] OR "comparative study"[Publication Type] OR "multicenter study"[Publication Type] OR "meta-analysis"[Publication Type] OR "systematic review"[Publication Type] OR "random allocation"[MeSH] OR "single-blind method"[MeSH] OR "double-blind method"[MeSH] OR "randomized controlled trials as topic"[MeSH] OR "cross-over studies"[MeSH] OR "matched-pair analysis"[MeSH] OR "placebos"[MeSH] OR "meta-analysis as topic"[MeSH] OR "systematic reviews as topic"[MeSH] OR random*[TIAB] OR rct[TIAB] OR "single blind*"[TIAB] OR "double blind*"[TIAB] OR "triple blind*"[TIAB] OR placebo*[TIAB] OR compar*[TIAB] OR "controlled stud*"[TIAB] OR "controlled trial*"[TIAB] OR "cross over*"[TIAB] OR crossover*[TIAB] OR "matched pair*"[TIAB] OR "clinical stud*"[TIAB] OR "clinical trial*"[TIAB] OR "multicenter stud*"[TIAB] OR "meta analys*"[TIAB] OR "systematic review*"[TIAB] OR "systematic overview*"[TIAB] OR metaanalys*[TIAB] OR "integrative research review*"[TIAB] OR "research integration*"[TIAB] or “one arm” or “single arm” or “Controlled Before-After Studies”[mesh] or “before and after study” |
| CENTRAL search conducted on 2021/7/28 n=144 |
| #1 MeSH descriptor: [Obsessive-Compulsive Disorder] explode all trees  #2 (OCD or obsessive-compulsive or obsessive　compulsive or obsess* or compulsi* or intrusi*):ti,ab,kw  #3 MeSH descriptor: [Self-Help Devices] explode all trees  #4 MeSH descriptor: [Computer Communication Networks] explode all trees  #5 MeSH descriptor: [Cell Phone] explode all trees  #6 MeSH descriptor: [Cell Phone Use] explode all trees  #7 MeSH descriptor: [Telemedicine] explode all trees  #8 MeSH descriptor: [Microcomputers] explode all trees  #9 (mHealth or telehealth or telemedicine or eHealth or m-health or e-health or telecommunication* or teleconference* or cellular phone* or cellular telephone* or cell phone* or mobile phone* or mobile telephone* or app or apps or application* or mobile* or smart* or wireless* or unguid* or self-direct or self-help self-manag* or self-act* or selfadmin or e-self-help or self-guid* or web or internet or online or web* or computer or eTherapy or e-therap*):ti,ab,kw  #10 MeSH descriptor: [Clinical Study] explode all trees  #11 #1 or #2 and (#3 or # or #5 or #6 or #7 or #8 or #9) and #10 |
| Embase search conducted on 2021/7/28 n=1464 |
| #1 'Obsessive-Compulsive Disorder'/exp OR 'OCD':ab,ti OR ‘obsessive-compulsive’:ab,ti OR ‘obsessive compulsive’:ab,ti  #2 ’self help device’/exp or ’computer communication networks’/exp or ’cell phones’/exp or ’cell phone use’/exp or ’telemedicine’/exp or ’Microcomputers’/exp or ‘mHealth’:ab,ti or ‘telehealth’:ab,ti or ‘telemedicine’:ab,ti or ‘eHealth’:ab,ti or ‘m-health’:ab,ti or ‘e-health’:ab,ti or ‘telecommunication*’:ab,ti or ‘teleconference*’:ab,ti or ‘cellular phone*’:ab,ti or ‘cellular telephone*’:ab,ti or ‘cell phone*’:ab,ti or ‘mobile phone*’:ab,ti or ‘mobile telephone*’:ab,ti or ‘app’:ab,ti or ‘apps’:ab,ti or ‘application*’:ab,ti or ‘mobile*’:ab,ti or ‘smart*’:ab,ti or ‘wireless*’:ab,ti or ‘unguid*’:ab,ti or ‘self-direct’:ab,ti or ‘self-help’:ab,ti or ‘self-manag*’:ab,ti or ‘self-act*’:ab,ti or ‘selfadmin’:ab,ti or ‘e-self-help’:ab,ti or ‘self-guid*’:ab,ti or ‘web’:ab,ti or ‘internet’:ab,ti or ‘online’:ab,ti or ‘web*’:ab,ti or ‘computer’:ab,ti or ‘eTherapy’:ab,ti or ‘e-therap*’:ab,ti  #3 ‘random allocation’/exp OR ’single-blind method’/exp OR ’double-blind method’/exp OR ’randomized controlled trials as topic’/exp OR ’cross-over studies’/exp OR ’matched pair analysis’/exp OR ’placebo’/exp OR ’before and after study’/exp OR ‘random*’:ab,ti OR ‘RCT’:ab,ti OR ‘single blind*’:ab,ti OR ‘double blind*’:ab,ti OR ‘triple blind*’:ab,ti OR ‘placebo*’:ab,ti OR ‘compar*’:ab,ti OR ‘clinical stud*’:ab,ti OR ‘clinical trial*’:ab,ti OR ‘cross over*’:ab,ti OR ‘crossover*’:ab,ti OR ‘matched pair*’:ab,ti or ‘before after study’:ab,ti or ‘before and after study’:ab,ti or ‘before-and-after’:ab,ti or ‘one arm’:ab,ti or ‘single arm’:ab,ti  #1 and #2 and #3 |
| ICTRP search on 2021/7/28 n=45 |
| Condition: Obsessive compulsive  Intervention: computer or digital or smart phone or smartphone or cell phone or mobile phone or internet or web or telemedicine or e-health or ehealth |
| ClinicalTrials.gov search n=395 |
| Condition or disease: Obsessive-Compulsive Disorder  Study type: Interventional studies(clinical trials) |
| Devices@FDA search on 2021/7/28 n=93 |
| Obsessive compulsive disorder |
