# Supplementary figures and images for: Unguided Computer-Assisted Self-Help Interventions Without Human Contact in Patients With Obsessive-Compulsive Disorder: Systematic Review and Meta-analysis
Source: J Med Internet Res. 2022 Apr 21;24(4):e35940. doi: 10.2196/35940 (PMC9073609; doi:10.2196/35940)

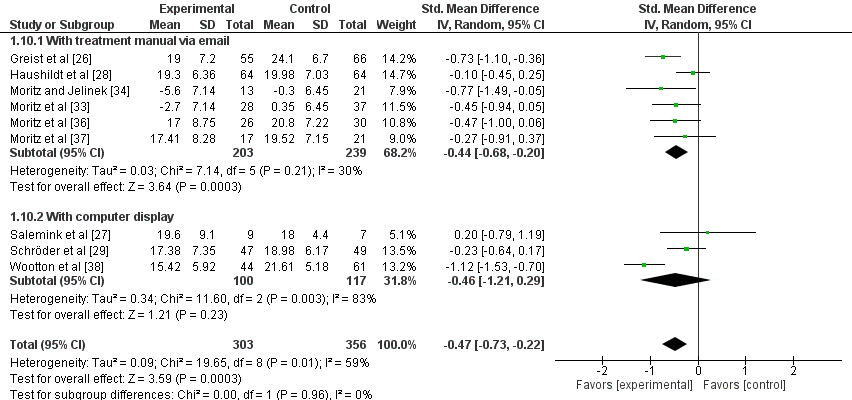

Supplement: Multimedia Appendix 3 [file jmir_v24i4e35940_app3.png]

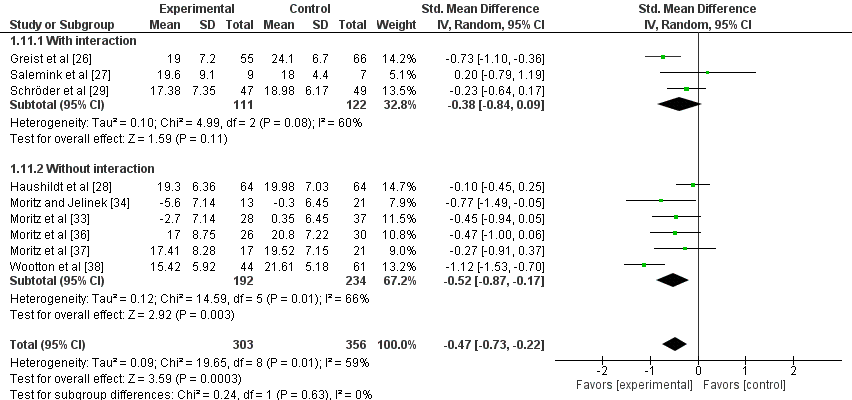

Supplement: Multimedia Appendix 4 [file jmir_v24i4e35940_app4.png]

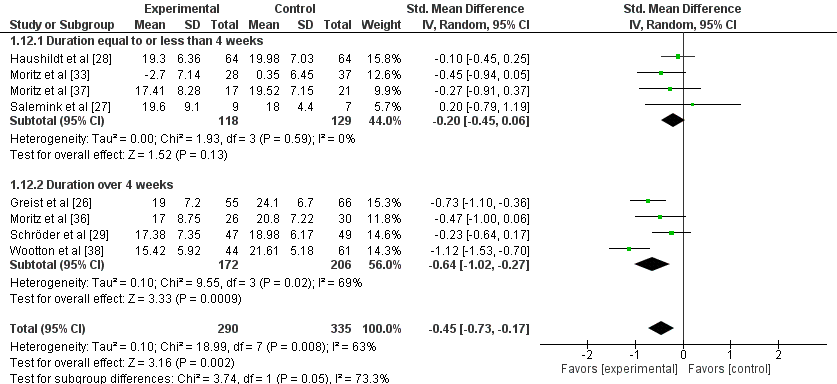

Supplement: Multimedia Appendix 5 [file jmir_v24i4e35940_app5.png]

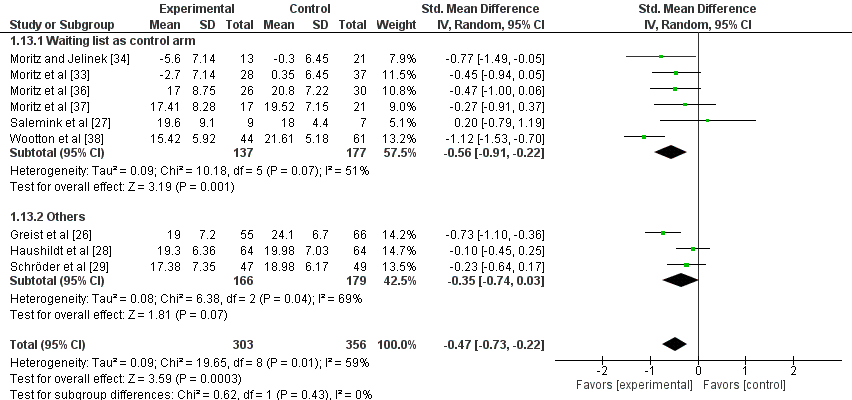

Supplement: Multimedia Appendix 6 [file jmir_v24i4e35940_app6.png]

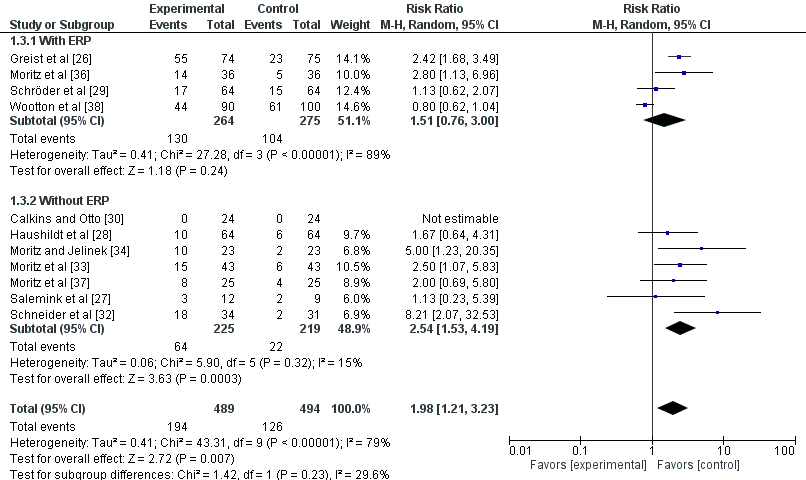

Supplement: Multimedia Appendix 7 [file jmir_v24i4e35940_app7.png]

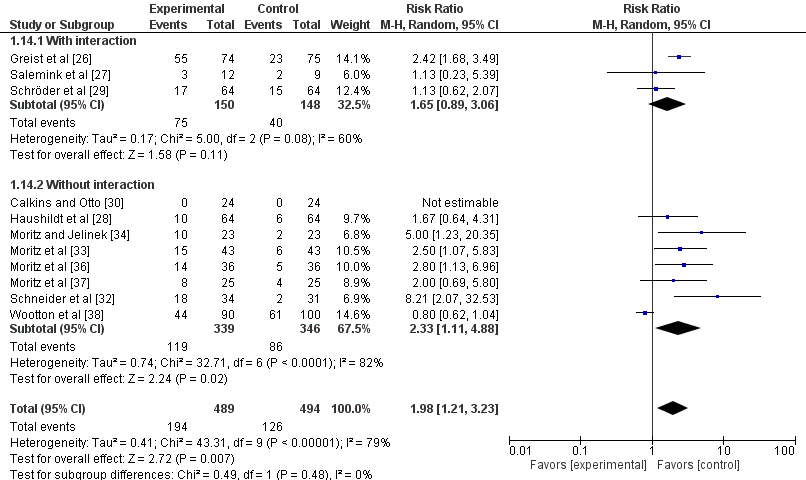

Supplement: Multimedia Appendix 8 [file jmir_v24i4e35940_app8.png]

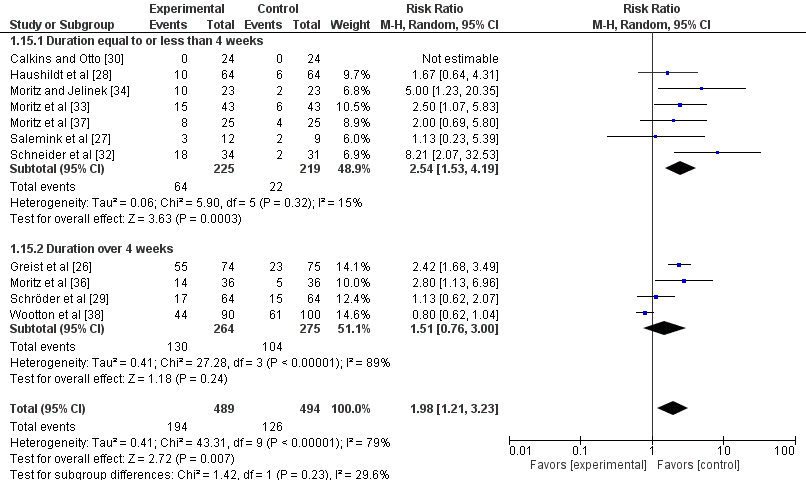

Supplement: Multimedia Appendix 9 [file jmir_v24i4e35940_app9.png]

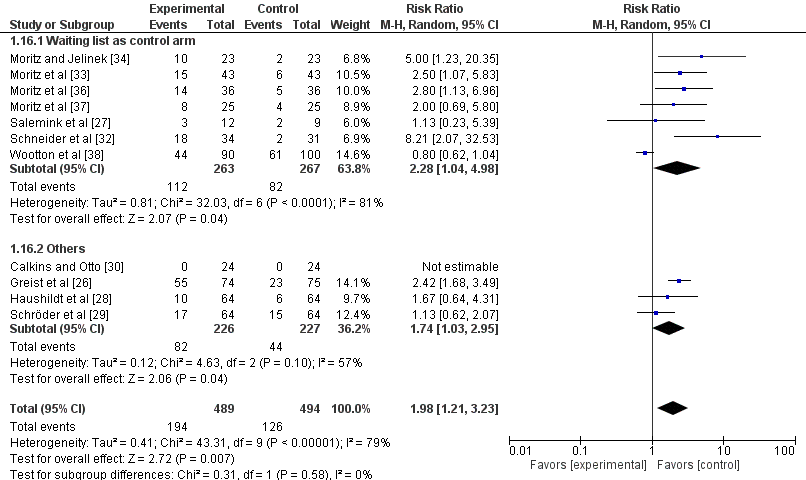

Supplement: Multimedia Appendix 10 [file jmir_v24i4e35940_app10.png]
